# Personalized Image-Guided Therapies for Local Malignencies: Interdisciplinary Options for Interventional Radiology and Interventional Radiotherapy

**DOI:** 10.3389/fonc.2021.616058

**Published:** 2021-04-01

**Authors:** Attila Kovács, Peter Bischoff, Hathal Haddad, György Kovács, Andreas Schaefer, Willi Zhou, Michael Pinkawa

**Affiliations:** ^1^ Clinic for Diagnostic and Interventional Radiology and Neuroradiology, MediClin Robert Janker Klinik, Bonn, Germany; ^2^ Clinic for Radiotherapy and Radiooncology, MediClin Robert Janker Klinik, Bonn, Germany; ^3^ Gemelli-INTERACTS, Università Cattolica del Sacro Cuore, Rome, Italy

**Keywords:** interventional oncology (IO), radiation oncology (RO), cancer immunotherapy (CI), abscopal effect, interstitial brachytherapy (ISBT), microwave ablation (MWA), electrochemotherapy (ECT), transarterial chemoembolization (TACE)

## Abstract

Minimal-invasive interventions considerably extend the therapeutic spectrum in oncology and open new dimensions in terms of survival, tolerability and patient-friendliness. Through the influence of image-guided interventions, many interdisciplinary therapy concepts have significantly evolved, and this process is by far not yet over. The rapid progression of minimal-invasive technologies offers hope for new therapeutic concepts in the short, medium and long term. Image-guided hybrid-technologies complement and even replace in selected cases classic surgery. In this newly begun era of immune-oncology, interdisciplinary collaboration and the focus on individualized and patient-friendly therapies are crucial.

## Introduction

After eight years, four times more patients who receive a combination of minimally invasive and systemic therapies survive compared to patients who receive systemic chemotherapy (SCT) alone. This significantly improved overall survival has been demonstrated in the CLOCC-trial, a randomized long-term study published in 2017, in patients with non-resectable colorectal cancer (CRC) liver metastases (1). Interventional oncology is the fastest developing area of interventional radiology. At the same time, minimally invasive, image-guided procedures (Minimal Invasive Therapies, MIT) are playing an increasingly important role in multimodal cancer therapy (1–3). In the last years, the concept of local tumor control has established itself as another pillar of modern oncology, not instead of, but complementary to the classical disciplines of systemic chemotherapy and surgery (1, 4). In addition to local tumor and symptom control, the proven immunomodulating effect of MIT will play an important, perhaps even a more decisive role than we suspect today, especially in the newly dawning age of checkpoint inhibitor therapy (2). The article *“How ablation destroys cancer to prolong lives”* from Aug 08, 2018 in *The Guardian* rightly asks the crucial question: *“So why is it not more widely known?”*

## Interventional Oncology

The oligometastatic paradigm hypothesizes that patients with a limited burden of metastases may achieve long-term disease control, survival benefit, or even cure, if the sites of disease can be removed. Although surgery was historically the primary modality used to remove metastases, newer and less-invasive modalities are now available, including stereotactic ablative radiotherapy (SABR) and image-guided ablations (4). Therapies are called minimally invasive if the success of the treatment is achieved in a way that is particularly gentle on the patient. The minimally invasive character of these interventions is often reflected in a non penetrative, or less invasive access route, so that many of these interventions can be performed on an outpatient basis or with a significantly reduced hospital stay. Instead of a large incision, at best punctures are required to target and destroy the respective lesion. In cancer therapy, a large number of interventions can already be carried out minimally invasively - unfortunately, experience shows that image-guided MIT are used far too rarely. The most common reason is that MIT are still rarely offered in the oncological therapy routine. Possible reasons for this may be due to the complexity of these therapies, which requires a particular specialization of the interventionalists, on the other hand also a close co-operation of the disciplines involved. In addition to the obvious advantages for the patients, these procedures present new challenges for the treating physician - one can neither directly see nor touch the tumor focus to be treated. Due to the limited exposure of the surgical site or the lack of manual palpation, important information is missing, which must be compensated by exact pretherapeutic planning. Radiologists, radiation therapists and nuclear medicine specialists are traditionally familiar with exactly this type of image-based diagnostics and image-guided therapy, i.e. with the creation of a “virtual” site. The core of this planning is an extremely precise imaging with recording of the anatomical, pathological and ideally also functional conditions - practically the creation of a virtual environment that is true to the millimeter. Modern imaging is required for therapy planning, as well as for controlling the intervention itself, documenting the results directly after treatment and, of course, for monitoring the progress of the treatment. Crucial for the success of these innovative therapies is the interaction of perfect imaging, constantly improved technologies and a great deal of experience of the therapist with so-called “keyhole procedures” – an emerging, but unstoppable advancing field of modern medicine. It is thus possible that ever more complex minimally invasive interventions in many areas of medicine are increasingly supplementing or even completely replacing classic surgical procedures. It is therefore advisable to first decide on an interdisciplinary basis and on the basis of imaging whether an interventional, a surgical or a combined procedure is to be used for the local tumor control. Hybrid interventions increasingly blur the line between surgery and intervention.

In contrast to alternative treatment methods, which could not achieve a survival benefit in the primary cancer therapy of various malignancies (compared to the guideline therapy, lung, colon and breast cancer showed a 2-fold, 4-fold and 5-fold increased risk of death with alternative therapies), MIT have fundamental advantages, which are particularly useful in cancer therapy (5). The motivation to use MIT is to support and complement it, to overcome the limitations of surgery and SCT and to improve the quality of life of patients.

Perhaps the most important advantage of MIT is that in combination with standard therapies it significantly increases overall survival (OS) compared to SCT alone. Two randomized-controlled trials (RCT) prove the evidence of OS advantage when using local ablative techniques (LAT) in combination with SCT versus SCT alone. According to the results of the CLOCC trial mOS at 8 years was significantly improved in the combined LAT and SCT-therapy arm versus the SCT-arm (36% versus 8%) (1). Analogous the SABR-COMET trial analyzed the impact of SABR in the treatment of different oligometastatic cancers (breast, lung, colorectal and prostate) (4). The 5-year OS was 42.3% in the combined therapy arm (SABR combined with standard-of-care SOC treatment) versus 17.7% in the SOC treatment arm. On the other hand, MIT have a good tolerability and without SCT-typical side effects such as hair loss, hand and foot syndrome, etc. SCT-associated chronic organ damages, like cardiomyopathy and sinusoidal injury, to name only a few, are a well-known limitation of systemic therapies (6, 7). Further restrictions are, that SCT has only limited effectiveness in many malignancies, e.g. renal cell carcinoma (NCC), cholangiocellular carcinoma (CCC) and hepatocellular carcinoma (HCC) and even newer drugs are only effective in defined subgroups – for example immunotherapy has proven effectiveness in the subgroup of pretreated mismatch-repair-deficient/microsatellite instable (MSI) CRC in stage IV – this makes up just 3-20% of the CRC patients (8, 9). Unresectable CRC liver metastases that are refractory to SCT benefit from liver-directed transarterial therapies (8, 10). Compared to classical surgery, the main advantages of MIT are the lower invasiveness, the lack of anesthesia, less pain and shorter hospital stays. In general, MIT have less impact on the quality of life and allow patients to spend more time in their family and professional environment while feeling comfortable (11, 12).

In order to take a closer look at the significance of minimally invasive procedures in cancer therapy, it is necessary to take an analytical view of the basic treatment approaches in oncology. For many solid cancers, surgical removal of the cancer is considered the gold standard for curative therapies. However, studies have shown that as long as the tumors are small enough, i.e. in their early stages, thermal ablation achieves similar results to surgery, but with the advantage that significantly less healthy tissue has to be sacrificed and that patients recover faster (13). This applies, for example, to liver cell cancer as well as to renal cell cancer. However, if a tumor is too large for surgical removal, chemotherapy is first administered to shrink the tumor to an operable size, so-called neoadjuvant therapy. Studies have shown, e.g. for metastases of colon cancer, that Transarterial Chemoembolization (TACE) achieves cytoreduction comparable to systemic chemotherapy, but with fewer systemic side effects (11, 14, 15). In case of multiorgan mestases it must be considered that not all metastases in all organs are life-limiting. In most cases it is the liver metastases that limit survival and should therefore be prioritized in the therapy. In the so-called oligometastasized situation, only a limited number of metastases are present in one or more organs. In this situation, it is common practice to treat metastases that are considered potentially dangerous in isolation and locally. This is the basic principle of radiotherapy, a generally accepted pillar of multimodal cancer therapy. However, analogue to radiation therapy, in some tumor entities and stages, at least comparable therapeutic results can also be achieved by MIT. Basic advantages of MIT are, that they have fewer systemic side effects compared to systemic chemotherapy and are tissue-sparing compared to classical surgery. A further advantage of MIT is its repeatability at one and the same localization in the event of local recurrence - an aspect that is limited for radiotherapy.

Studies have shown that especially in oligometastasized situations and a less aggressive tumor biology, the use of minimally invasive procedures can significantly extend overall survival with a good quality of life - sometimes by years (1, 4). MIT are taken into consideration by many oncologists only in the salvage situation. Unfortunately, in advanced tumor stages, even MIT has no positive effect on survival.

## Minimally Invasive Techniques

**Figure 1:**
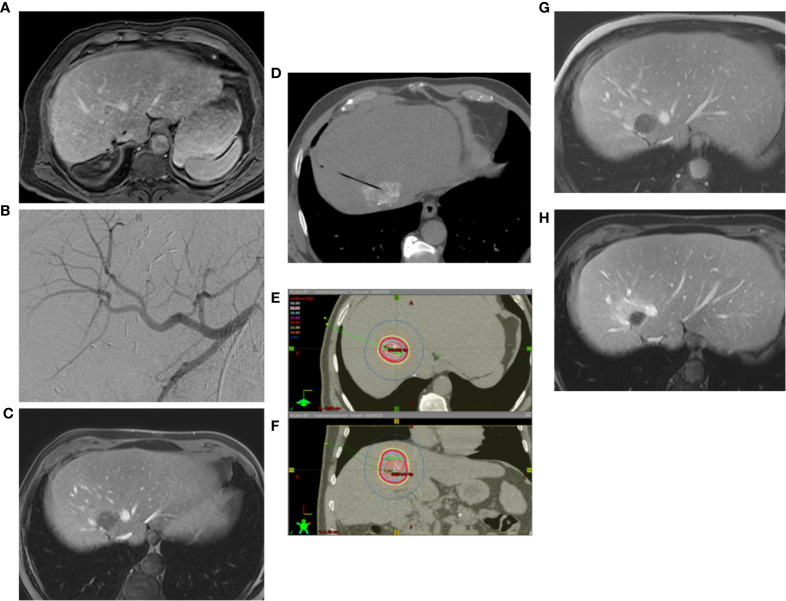
**(A)** Renewed solitary CRC-metastasis after multiple surgical metastasectomies, sometimes with complicated postoperative course until sepsis. **(B)** The difficultly located new metastasis between the hepatic veins in S VIII has been initially transartelically chemoembolized (TACE). **(C)** Post-interventional contrast-enhanced MRI shows subtotal devascularisation of the target lesion. **(D)** Interstitial brachytherapy was performed sequentially. Lipiodol labelling from TACE was used for navigation of the brachytherapy applicator. **(E)** The image shows the isodose distribution in the axial plane. **(F)** Isodose distribution in the coronary plane. **(G)** contrast enhanced MRI reveals an excellent local tumor control after 3 months. **(H)** After 6 months the tumor cavity shrinks in time, there is still no recurrence, only perifocal postradiogenic changes.

**Figure 2:**
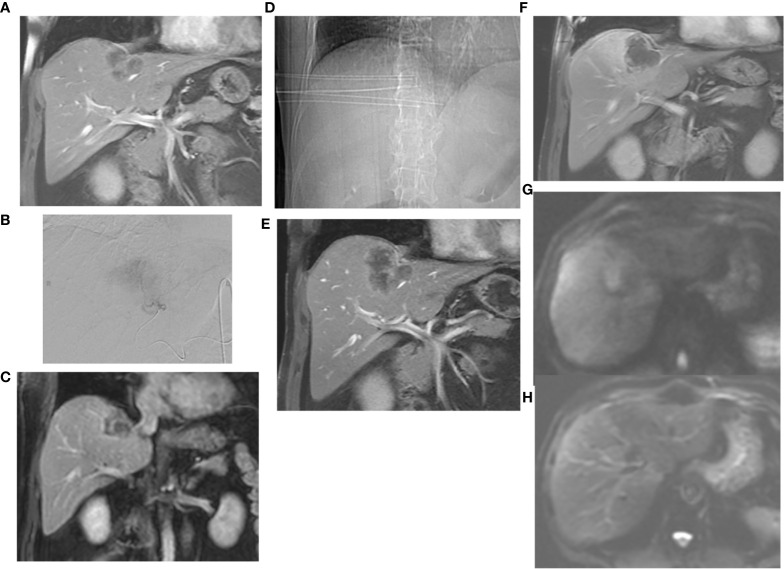
**(A)** Under third-line systemic chemotherapy, progressive solitary, surgically unresectable colorectal liver metastasis on the border between segment IVa and VIII. **(B)** The lesion has been initially transarterially chemoembolized with DEB-IRI (Irinotecan-loaded drug-eluting beads). **(C)** contrast-enhanced MRI shows subtotal devascularisation of the metastasis with a still vital tumour margin on the right lateral-apical side. **(D)** The marginal recurrences have been interstitially brachytherapied in the interval. The topogram clearly shows the parallel positioning of the applicators. **(E)** Excellent local tumor control was observed after 3 months. **(F)** timely shrinkage of the metastasis as well as further local tumor control without detection of recurrence after 6 months. **(G)** Local tumor control also confirmed after 12 months by the absence of diffusion restriction in DWI. **(H)** The corresponding ADC-maps.

The primary goal of minimally invasive, loco-regional therapies is to destroy primary and secondary malignancies efficiently, simultaneously and gently using imaging techniques. Interventional therapies are divided into percutaneous and endovascular procedures. In percutaneous procedures, the tumor is accessed through the skin. In most cases, a 1-2 millimeter small puncture is sufficient to insert the instruments. Until today, different methods have been established to destroy the tumor locally, e.g. heat up to 170° or cold down to -100°. Depending on the procedure, the heat is achieved with alternating current (radio frequency ablation, RFA), or microwave (MWA), or by bundled ultrasonic waves (HiFu). In the case of cold therapy (cryoablation) by local icing. Depending on the technique, the probes work independently in standalone mode, as in the long-established radiofrequency ablation (RFA), which uses alternating current to heat the tumor tissue. A disadvantage of RFA is the therapeutic limitation to smaller target lesions up to 3.5 cm in diameter. Other techniques can synchronize the delivered energy of several probes with each other, so that the ablation zone can be enlarged, in the case of microwave and cryoablation for example up to 5 cm tumor diameter. A special form of local therapy is internal radiation (interstitial brachytherapy), which uses neither heat nor cold, but radiation with a very limited range (**Figure 1**). The size and configuration of the target region as well as the radiation sensitivity of the surrounding organs can be adjusted by several applicators and a very precise “dose-painting”. All of these therapies cannot be considered equivalent, because not all therapies are equally successful for all types of cancer, lesion sizes and localizations. Therefore, the specialist for interventional procedures must decide individually for each patient, each type of tumor and each localization - ultimately for each individual target lesion, which therapy is the optimal one. In endovascular procedures, the cancer-supplying arteries are precisely targeted by a microcatheter. This gives the possibility to apply drugs directly into the tumor (**Figure 2**). To ensure that the medication remains in the tumor and is not flushed out, the drugs are bound to small beads of a few micrometers in size (TACE = Transarterial Chemoembolization). This has two advantages: on the one hand, the blood supply to the tumor is reduced or completely cut off. This alone causes the cancer cells to begin dying off and opens their cell walls so that the drugs can penetrate more easily. The second advantage is that the globules only release the drugs in the tumor, so that they are not distributed throughout the entire body and thus do not affect the entire body. In addition, embolics allow a slower release of the drug, up to two weeks, so that step by step all cancer cells are captured by the drug. Therefore, in most tumors, regardless of whether percutaneously or endovascularly treated, a complete destruction of the treated cancer cells can be detected after only a few days.

Despite this diversity of thermo- and radioablative techniques, these procedures are limited in certain situations. These include target lesions whose size exceeds the safe ablation zone of thermal procedures. Thermal procedures are also affected by the “heat-sink” effect, i.e. the undesired cooling of heat probes near vessels; this effect is particularly pronounced in RFA. The development of thermal necrosis is decisively influenced by the thermal resistance of the tissue. Radio ablation is again limited in the vicinity of radiation-sensitive organs. In such cases chemoablation is a welcome addition to the portfolio of local therapies. Electrochemotherapy (ECT) is a combined tumor therapy that enhances the local effect of a systemically administered chemotherapeutic agent by reversible electroporation. In contrast to the mostly thermal ablative procedures, ECT is a cytotoxic local ablative therapy, which is mediated by electrical impulses. The electrical impulses are used to temporarily increase the permeability of the tumor cell membrane and thus promote the entry of the cytotoxic agent into the cell. This is essential for chemotherapeutic agents with large and complex molecular structures, such as bleomycin, which otherwise could not enter the tumor cells. Bleomycin as a cytostatic drug is composed of two single compounds, bleomycin A2 and B2, each with the molecular formula C55H84N17O21S3+ and C55H84N20O21S2. Thus, bleomycin, with a molar mass of around 3000 g/mol, is considered a heavyweight compared to other standard oncological therapeutics. ECT opens the cell membrane not only for bleomycin, but also for other poorly permeable cytostatic drugs. The resulting increase in efficacy varies depending on the cytostatic drug and is a factor of up to 700 for bleomycin. A major advantage of bleomycin is its toxicity independent of histology, i.e. its efficacy is largely independent of the underlying tumor entity. ECT has already been shown to be effective in primary and secondary skin tumors. The procedure has the advantage of a response rate of 70-80% and hardly damages surrounding tissue. ECT seems to be particularly effective in basal cell carcinoma, where the therapy led to a complete remission in 91% of cases (16). ECT is also convincing in the case of metastases in parenchymatous organs. One study has shown an 85% complete response, or 15% partial response, of liver metastases from mCRC one month after ECT, and 71% and 29% 5 months after ECT, respectively (17). The individual metastases were up to 29mm in diameter and 48% were in the immediate vicinity of large vessels. ECT is a non-thermal local ablation technique with some advantageous features. Bleomycin as an effective agent exhibits high toxicity against a variety of tumor entities. Despite its therapeutic effectiveness, collagenous structures such as vessels and bile ducts are spared. ECT is repeatable and suitable as a local therapy intermittently between chemotherapy cycles. ECT has the potential to close relevant gaps in local ablative therapy: e.g. in lesions that are too large for thermal ablation, in non-radiation-sensitive tumors or if the target lesion is located in the immediate vicinity of radiation-vulnerable organs. Patients experience ECT as a well-tolerated, painless therapy with few side effects and no relevant pain, nausea or systemic side effects. 

## Evidence

In the oligometastasized situation, minimally invasive interventions should be used as early as possible to achieve a significant survival advantage, to preserve organ reserves and, last but not least, not to impair the quality of life (18). MIT achieve comparable results as surgery for tumors discovered early. For example thermal ablation achieves in small renal cell carcinomas comparable oncological results to partial nephrectomy, but is associated with less collateral damage, such as limitation of the glomerular filtration rate, blood loss and hospitalization (19). In general, minimally invasive procedures better preserve the functional reserve of the treated organs and are also health economically more advantageous compared to surgical procedures. Last but not least, MIT are also relevant in the context of demographic developments. On the one hand, the patient clientele is getting older and older, on the other hand, patients with malignant diseases survive longer and longer due to early detection and improved therapies. Accompanying diseases, which naturally increase in frequency with age, often limit aggressive therapies. One advantage of minimally invasive radiological procedures is that tumors can be destroyed without affecting the entire organism and the surrounding healthy structures. These procedures generally have few side effects and are gentle on the organs, which is why they are also suitable for elderly patients with concomitant diseases. In the palliative situation, the best possible quality of life for the patient is just as important an objective as the long-term control of the tumor. A particularly important and frequently affected organ in the overall context of oncological diseases is the liver, which must also be prioritized accordingly. Besides primary liver malignancies, such as hepatocellular carcinoma (HCC), secondary liver malignancies, i.e. metastases, play an increasingly important role and are an important treatment goal of local therapies. Liver metastases are of prognostic relevance and should also be prioritized in the case of synchronous extrahepatic tumor manifestation, since the latter are, with a few exceptions, not life-limiting. The above also applies to the liver, that not every method is equally suitable for all lesions. The choice of therapy is determined by the number, size, configuration and location or environment of the target lesion. Thus, the various thermo-, radio- and chemoablative procedures do not compete with each other, but complement each other and are used as a supplement in the hands of the experienced interventionalist. The radiological-interventional expertise therefore implies not only the experience of the therapist, but also that a broad spectrum of procedures and technologies must be available, which can then be used in an optimized way to meet individual requirements.

In discussing the available evidence, we focus on metastatic colorectal carcinoma (mCRC) on the one hand because of its high incidence, and on the other hand because of the comparatively rapid new developments in recent years compared to the majority of other tumor entities.

Colorectal cancer (CRC) is one of the most common malignant diseases. In recent years, the clinical outcome of patients with metastatic CRC has improved significantly. This is due to improved surgical techniques, improved chemotherapeutic agents, and an expansion in the use of ablative techniques. For this purpose, the entire “toolbox” of local therapeutic procedures must be known and available and must be discussed on an interdisciplinary basis. Oligometastatic disease (OMD) is defined as a stage between limited local tumor and extensive distant metastasis. When a mCRC is to be considered “palliative” has changed in all areas of oncology in the course of further development in recent decades. The therapeutic strategy for OMD is based on the possibility of a complete reduction of all tumor masses, that can improve the clinical outcome of patients. OMD means a metastasis limited to a few organs (1,2,3) and lesions (<5) in resected or resectable primary tumor. The aim of the individual therapy sequence, which is decided upon within the framework of an interdisciplinary tumor conference, is to assess whether the disease is primarily resectable or non-resectable or, after prior treatment, potentially resectable. The prerequisites for an optimal decision are adequate imaging and the performance status of the patient. Molecular aspects of the tumor provide additional information on specific treatment prospects and prognosis. The aim of all considerations is to identify patients with a comparatively less aggressive tumor biology who will benefit from a localized intervention, possibly in combination with a systemic therapy. At presentation, 20-25% of patients will have distant metastases, most to the liver. Another 20-25% will later develop liver metastases. 49% will have a liver dominant disease, and 83% will have some liver involvement. Disease specific survival is also significantly shorter for those who die of liver metastasis, compared to patients who die of other metastatic sites. In CRC the liver is the most frequent site of metastases and dominates the length of survival. For a patient with a resectable solitary colorectal liver metastasis (CRC-LM), surgery offers a clear and significant benefit in terms of long-term survival. Patients with limited LM have a survival advantage even after multiple liver resection procedures, and 5-year survival rates of more than 40% are achieved in a multimodal approach. The fact that liver resection is affecting outcome is also highlighted by the fact that over 70% of the patients with unresectable liver metastases die of their liver metastases. But also in patients treated by hepatectomy, 30% ultimately die of liver metastases. It should also be noted that 70% of the patients who receive neoadjuvant chemotherapy and have so called “disappearing liver metastases” will have microscopic residual foci in the liver, which is site of local recurrence in 60% of these patients. Thus, addressing liver metastases initially is the most clinically relevant, since this is the most life limiting. As such, liver directed therapies shift the cause of death to other sites at a later time point (20). Although the individualization and personalization of oncological therapy for CRC has not yet been included in guideline recommendations, an individual risk profile should be established for each patient in the future based on molecular markers and clinical tumor characteristics and should be taken into account when deciding for or against maximally invasive resection procedures (e.g. associating liver partition and portal vein ligation for staged hepatectomy) or other local ablative measures. It is difficult to predict the long-term success of local therapy of oligo- metastases based on clinical or molecular tumor characteristics. Therefore, in case of suitable patients, the local therapy will always be the best choice. On the available regional treatment approaches for CRC-LM include

- surgical resection,
- thermal ablation,
- regional intraarterial chemotherapy of the liver,
- Chemoembolization,
- Radioembolization and
- Radiotherapy (RT), including stereotactic RT (“stereotactic body radiation therapy”, SBRT) and “interventional brachytherapy”, IBT).

This armamentarium of local therapy procedures is also known as so-called ESMO-Toolbox. It is the task of the treating surgeons, oncologists, radiotherapists and interventional radiologists, together in an individual approach to the individual patients´ best therapy sequence (first systemic or first locally) and the best therapy modality (systemic and/or local). This interdisciplinarity is indispensable, because foreign disciplines cannot adequately judge the potential of the individual local procedures in an expert manner (21, 22). In a study for resectability assessment in easily resectable disease as classified by specialized surgeons, among oncologists only 34% of the cases have been found to be resectable (23). Thus also large differences in the frequency of referrals for liver resection was found, whereby a 10-fold variation between the centers with the highest and lowest transfer rate is reported (24). In Germany the target value of >10% secondary LM resections even in the cancer centers certified by the German Cancer Society intestinal centers are only reached by two thirds. Amazingly enough even in patients with a singular metastasis, only in 52% of cases a liver resection (25). In summary despite significant improvement in the probability of survival after local therapies are still too few patients in specialized centers were presented. The timing of the local intervention after neoadjuvant chemotherapy should also be well planned. Neoadjuvant treatment strategies cause time-dependent liver e.g. by sinusoidal obstruction syndrome after oxaliplatin-based chemotherapy and steatohepatitis with higher rates of infectious complications after irinotecan-based chemotherapy and lead to an increased 90-day mortality liver failure after surgery (26).

Due to the size and localization of metastases or because of the general condition, however, more than four-fifths of the patients has an inoperable disease. Various non-surgical, ablative options are available, which are just as resection techniques and the effectiveness of systemic therapies constantly improving. It is common that the choice of ablation technique often depends on the institution and specialty. For the best individual result, however, it would be more advantageous if all or at least several common techniques were available in order to select the best possible alternative for the respective case. This is certainly more expensive to maintain but would have the advantage of broadening the range of treatments and possibly reducing the local recurrence rate. Continuing this train of thought, the same applies to the combination of several local procedures, each of which is well tolerated but which can in sum increase local tumor control. Patients with non-resectable colorectal liver metastases are an important group of patients who benefit significantly from local therapies. The CLOCC study, a randomized long-term study published in 2017, showed a significantly improved overall survival for patients receiving a combination therapy of local ablation procedures and systemic chemotherapy compared to chemotherapy alone (1). One of the key findings of the CLOCC study is that 4 times more patients survived in the combined therapy arm after 8 years than in the chemotherapy arm alone. Taking these impressive data into account, the European Society of Medical Oncology (ESMO) has responded by including local ablation procedures in the current consensus paper on the treatment of metastatic colorectal cancer (mCRC) (27). The ESMO guidelines even allow a high degree of flexibility in the choice of thermal ablation methods: “A treatment goal of ablation is a relatively new concept for patients with mCRC and involves an attempt to eradicate all visible metastatic lesions using the best instrument from the toolbox of LATs (abbreviated as Local Ablative Therapies), in combination with systemic therapy”.

External beam radiation therapy (RT) plays only a limited role in the treatment of LM due to the high rates of radiation-induced liver disease (“radiation-induced liver disease”, RILD) when a large percentage of the liver is exposed to the radiation dose. With advances in treatment, image guidance and motion control it is possible to administer ablative radiation doses while sparing the rest of the liver. For patients with CRC-LM the SBRT has proven to be effective. Low toxicity rates have been reported and RILD are rarely described after SBRT in non-cirrhotic patients (28, 29). The largest series of long-term follow-ups for SBRT in CRC-LM reported 65 patients with 102 lesions (30). The overall rate of local control was 71%, with patients with higher biologically equivalent dose from ≥79 BED, a local control rate of 86%, 80% and 71% after 12, 18 and 24 months in the past. In terms of toxicity, almost 20% of patients showed higher levels of gastrointestinal toxicity or liver enzymes. Additionally, mature monoinstitutional experiences with IRT demonstrated the advantage of focal high-dose-rate interstitial radiotherapy in effectivity and economics (31–35).

Endovascular therapies (EVT) should be used in cases of liver-dominant metastasis, and be considered, to be carried out when a first or second line therapy is progressive or shows residual metastasis after systemic therapy. EVT are preferable to ablation and SBRT if in a liver lobe several LM are present, which can be treated simultaneously. In comparison, transarterial chemoembolization (TACE) of colorectal liver metastases (DEBIRI) showed not only a survival benefit but also better tolerability compared to intravenous systemic therapy (FOLFIRI) (10, 11).

There is little evidence of perioperative or periinterventional Chemotherapy for local therapy of OM. The expectation that patients with a lower-risk oncological disease would benefit significantly less from perioperative chemotherapy was confirmed by a large retrospective study involving almost 1500 patients with solitary, resectable metastases of the CRC further confirmed. The study compared patients who received at least 3 cycles of systemic therapy with those who underwent only surgery. The rate of post-operative complications was in the chemotherapy group significantly higher (37.2% vs. 24%, p= 0.006), without overall survival improved (36). Probably have only patients with a medium and high oncological risk benefits of systemic therapy.

In summary, it is important to evaluate any patient with mCRC initially and then at regular intervals repeatedly in an interdisciplinary tumor board for the most promising individual therapy in particular the sequence between local therapy.

A limitation of the currently available evidence is that the above-mentioned techniques have been investigated alone or in combination with established systemic and surgical therapies, on the other hand, the combination of interventional-radiological and radiotherapeutic procedures is limited to individual case reports. Combined minimally invasive and radiotherapeutic interventions are currently and in the near future will be reserved for dedicated centers that have the expertise and logistics to cooperate. The European CanCer Organisation (ECCO) defines the essential requirements for quality cancer care as “challenges, organization and actions that are necessary to give high-quality care to patients who have a specific type of cancer” (37). The shift in modern oncology towards personalized medicine is an extremely welcome development. Although both terms are often used synonymously, the distinction between precision medicine, which is directed against individual gene mutations, responsible for the development and growth of a specific tumor, and personalized medicine, which stands for a holistic view of the individual constellation and involves the patient as an equal partner in the decision-making process, is essential. The Delphi-study, published 2019, assessed the relevance and the implementation of patient-centeredness (PC) from the patient´s perspective in Germany (38). The results of the study paint a worrying picture: many physicians make decisions without openly discussing treatment alternatives with their patients, even though the interests of their patients are actually very important to them. All dimensions of PC (e.g. uniqueness of each patient, consideration of personal circumstances, teamwork of healthcare providers and collaboration as equal partners and involvement in decision making, to name some of the 15 points) considered by the patients to be relevant, were not well implemented. The authors concluded that these findings should not be neglected and further policy makers and other stakeholders, interested in fostering PC healthcare should focus on a wholesome perspective considering the patients´ rating. It is no longer just a requirement of various guidelines, but also a general practice that the treatment strategy for every cancer patient must be determined and carried out by multidisciplinary tumor boards (MDT) consisting of medical, surgical and radiation oncologists, diagnostic and interventional radiologists, nuclear medicine specialists and pathologists (39). Studies on decision-making in multidisciplinary cancer team meetings (MDTM) came to a similar result. In general, MDTMs were not in line with the principles of PC care (40). Studies that have investigated the inclusion of patient perspectives in joint decision-making confirm that MDTM do not exhibit shared decision-making (SDM). Patient perspectives are in general absent and the entire decision-making process do not follow the principles of SDM (41, 42). The authors conclude, if MDTM wish to become more patient-centered they will have to modify their processes and find a way to include patient preferences into the decision-making process. Medicine has become more complex overall. Today there are significantly more treatment options available than in the past. Knowledge about the opportunities and risks of the various therapies is constantly growing and poses the dilemma that it is not always clear whether treatment X is better than treatment Y in a specific case. In the future, doctors and patients will share the challenge of finding the best solution in each case.

## Future Prospects

Local-ablative procedures are able to achieve survival rates such as resection, but the latter remain superior in terms of local recurrence rate. Multidisciplinary treatment decision and performance in dedicated expert centers offers the best possible results (22).

Immunotherapies not only enrich the therapeutic spectrum in drug oncology, but also make a permanent activation of the immune system with the goal of a long, chemotherapy-free control of the disease seem possible. However, immunotherapies are currently still limited due to their tolerability and the accessibility of individual tumors. The deciphering of tumor and immune system stimulating or suppressing mechanisms will decisively determine oncology in the near future. The success of cancer immunotherapy has generated a tremendous interest in further developing and exploring strategies in combination with other approaches such as radiotherapy and local ablative therapies (43). The future perspectives of local therapies indicate an exciting development beyond the mere local tissue destruction and local tumor control. Research is currently underway to make tumors accessible for immunotherapy – to convert immunologic “cold” tumors into responsive “hot” tumors – through interventional priming or to apply immunotherapeutics locally (9). The already proven possibility to generate a kind of cancer vaccination by physical tumor destruction, which in turn enhances the therapy with checkpoint inhibitors and thus the abscopal effect, is a completely new motivation for the use of local ablative procedures (2, 44). Access to the tumor and its microenvironment are key pillars of immunotherapy. MIT can expose tumor debris for sensing immune response in nearby tissue and lymph nodes, thus activating T cells to fight cancer. It seems that even various MIT are able to release a broad spectrum of polyvalent tumor antigens from the entirety of heterogeneous tumor cell populations - in the sense of an in-situ anti-cancer vaccination. Our challenges are to build the evidence to integrate MIT into the overall treatment of cancer in various therapeutic sequences – induction, combination or adjuvant – with systemic therapies (45). These findings as well as future developments could soon lead to a paradigm shift in oncological therapy. We can look forward with excitement to the developments in the coming years, where local procedures will no longer be considered competitive to standard surgical-oncological therapies, but will be used as adjunct to immunotherapy.

## Conclusion

The era of personalized medicine presents a great challenge and opportunity for cancer imaging and therapy. Interventional radiology and radiotherapy have a long history of innovation in minimally invasive image-guided procedures. Patient-specific therapies are increasingly replacing standard histology and organ-based algorithms (46). The early recognition of the biology and spacial heterogeneity of tumors will aid appropriate selection of therapy according to molecular profile. Supported by strong basic and clinical research MIT significantly expand the therapeutic spectrum in oncology, which unfortunately remains hidden from most therapists who are not in discussion with interventional therapists. MIT are rarely considered in guidelines. This is more than regrettable and not only detrimental to patients, but also to health economics. Minimally invasive medicine opens up new therapeutic dimensions and is to be regarded as one of the protagonists of innovative modern medicine, perhaps even as a barometer of the future viability of our health system. It is generally undisputed that optimal therapeutic results in oncology can only be achieved through interdisciplinary concepts that exploit all local, locoregional and systemic therapeutic options beyond the boundaries of individual disciplines. Many treatment concepts have changed significantly under the influence of minimally invasive procedures and this process is not yet complete. The rapid development of minimally invasive therapies gives hope for new therapeutic concepts in the short, medium and long term. In times of hybrid technologies and immunoncology, a new culture of interdisciplinary cooperation of therapists and the concentration on individualized and patient-oriented therapies is more than desirable. As today´s cancer cure requires a multidisciplinary approach, treatment combination is thus a far more pressing concern than treatment competition. Our concrete recommendation for the here and now: we can only encourage patients and therapists to get a second opinion in a specialized clinic for MIT including interventional radiology and radiotherapy.

## Data Availability Statement

The raw data supporting the conclusions of this article will be made available by the authors, without undue reservation.

## Author Contributions

AK wrote the paper. PB, HH, AS, WZ, and MP contributed to the data acquisition. GK supported the team in an advisory capacity and proofread the paper. All authors contributed to the article and approved the submitted version.

## Conflict of Interest

The authors declare that the research was conducted in the absence of any commercial or financial relationships that could be construed as a potential conflict of interest.
